# Editorial: Editors’ showcase 2023: insights in cell adhesion and migration

**DOI:** 10.3389/fcell.2024.1497689

**Published:** 2024-10-03

**Authors:** Arie Horowitz, Akiko Mammoto, Vladimir Sytnyk, Igor Jakovcevski

**Affiliations:** ^1^ Department of Biomedical Engineering, The University of Texas at Austin, Austin, TX, United States; ^2^ Department of Pediatrics, Medical College of Wisconsin, Milwaukee, WI, United States; ^3^ School of Biotech and Biomolecular Science, University of New South Wales, Kensington, NSW, Australia; ^4^ Department of Human Medicine, Faculty of Health, Witten-Herdecke University, Witten-Herdecke, Germany

**Keywords:** mechanotransduction, extracellular matrix, actin, myosin-2, stress fiber, focal adhesion, mesenchymal, amoeboid

Adhesion, whether to the extracellular matrix (ECM) (Saraswathibhatla et al., 2023) or to adjacent cells (Friedl and Mayor, 2017; Mayor and Etienne-Manneville, 2016) is essential for cell migration. This dependence is the focus of the current Research Topic, comprising six reviews and two original research papers. Cell movement occurs in multicellular organisms both singly and collectively: The first category consists primarily of immune system cells traversing the vasculature and extravasating into the surrounding tissue (Kameritsch and Renkawitz, 2020) or malignant cells that follow a similar pattern (Paul et al., 2017). Collective cell movement occurs from the earliest steps of oocyte gastrulation to tissue morphogenesis in the maturing organism (Scarpa and Mayor, 2016).

Cell migration involves cytoskeleton remodeling (Blanchoin et al., 2014), molecular motor activity (Vicente-Manzanares et al., 2009), membrane trafficking (Maritzen et al., 2015; Wilson et al., 2018), and displacement of the nucleus (Calero-Cuenca et al., 2018). Unless propelled by their own flagella (Leung et al., 2021), or by peristaltic swimming (Martin et al., 2020), cells must generate traction by homotypic or heterotypic binding of surface proteins to their counterparts on the surface of adjacent neighbors (Venhuizen and Zegers, 2017), or extracellular matrix proteins (Pally and Naba, 2024). Migration frequently occurs along chemoattractant gradients that are recognized by surface receptors (Insall, 2023).

Three of the reviews address recent progress in the quantification of cell migration and highlight the advantages of either limiting the cells’ degree of spatial freedom versus maintaining 3D reality. Toscano et al. present an exhaustive analysis of the growing number of applications and plugins for the quantification of multiple cell parameters, from the morphology of single cells to collective cell movement in 2D. Their Table 1 is a resource of computer applications. Some applications employ interactive machine learning for object classification and 3D movement analysis (Berg et al., 2019), a likely method of choice because of its versatility and flexibility. Heyn et al. use a reductionist approach of limiting cell movement to a single trajectory to simplify and standardize the identification of cellular dynamics and to acquire sufficient data for testing explanatory biophysical models. This is suitable for studying adhesion-dependent migration of mesenchymal cells. Cell dynamics are modeled by a non-linear spring-like constitutive relationship between cytoplasmic contractile forces and static bonds to the extracellular matrix (ECM). The authors invoke this ‘clutch mechanism’ to account for a universal correlation between the cell speed and movement persistence. While the merits of 1D are compelling, it is conceivable that confinement suppresses activities that require 3D to appear. The extensive literature cited by Toscano et al. is a convenient resource on 1D cell motility.

Rodríguez-Cruz et al. studied cell migration in 3D collagen-1 gels to determine the relationship between ECM density and mammary cancer cell invasion. Within the limited 6-fold range of collagen concentrations, invasion distance decreased only at the highest (6 mg/mL) concentration. Their round shape and a peripheral filamentous actin band indicate that cells encapsulated in 6 mg/mL gel transition from a mesenchymal to an amoeboid phenotype. Though no mechanism is invoked to drive the transition, the authors suggest that the ameboid phenotypes is selected in cancer, expediting tumor invasion.

Though the reviews of Katsuta et al. and of Estrach et al. focus on focal adhesions and the ECM, respectively, both address the effects of force transduction between these mechanically coupled systems. The former study attributes a central role in tensile forces sensing to focal adhesions and the stress fibers they anchor. Actin crosslinking proteins, including α-actinin, filamin, and non-muscle myosin-2, are purported to constitute the force-sensing molecular mechanism, though their mode of action is not specified. Presumably, force-generating myosin-2 has a more pronounced role than the non-catalytic crosslinkers, as suggested by a study the authors cited (Raab et al., 2012). Estrach et al. address the wider role of the ECM in maintaining the epithelial cell phenotype in the gut, lung, and skin, whereby transduction of mechanical stimuli through integrin to the actin cytoskeleton and sequential activation of Src, focal adhesion kinase (FAK) activates the transcriptional activity of YAP1/TAZ. An interesting exception to this pathway is the epigenetic modification caused by deformation of the nuclear envelope when cells squeeze through narrow pores in dense ECM, of the type studied by Rodríguez-Cruz et al. (ibid.).

The review of Buffone et al. and the study of Mellentine et al. address directed cell migration, albeit of single ameboid cells versus collective cell migration through a different mechanism. The former discusses leukocyte upstream cell motility, i.e., against the shear force exerted by blood flow. Upstream migration is thought to facilitate leukocyte movement to the origin of the chemotactic signal and/or to sites on the lumen that are amenable to transmigration through the vessel wall. It is mediated by integrin α_L_β_2_ binding to endothelial ICAM1. ICAM1-bound integrin α_L_β_2_ transduces the signal through the Crk adaptor protein. Since Crk participates in Rac1 activation (Kiyokawa et al., 1998), it may induce actin polymerization in the lamellipodium (Lawson and Ridley, 2018). Mellentine et al. focus on prostaglandin (PG)-induced collective cell migration in the prototypical model of the *Drosophila melanogaster* ovary (Pocha and Montell, 2014). While the role of PG in border cell migration is known, it is unclear whether it affects solely border cells or also the surrounding nurse cells. The study dissects elegantly the PGs specific roles. The PGs have two cell type specific functions: PGE_2_ acts on nurse cells while PGF_2α_, acts on border cells, sustaining border cell migration; PGF_2α_, acts on border cells, promoting their clustering. The authors propose that the two PGs sustain migration by inhibiting myosin-2, thus reducing the stiffness of both cell types, whereas PGF_2α_, is required for the cell membrane localization of integrin, without specifying the respective mechanisms. Images of the phosphorylated myosin-2 light chain, a myosin-2 activation marker, suggest that, in the absence of PGs, both the localization and the activity level of myosin-2 are dysregulated. The applicability of PG-dependent collective cell migration to vertebrates remains to be investigated.

The review by Arabi et al. differs from the rest in that it does not address cell adhesion or migration *per se*. Rather, it discusses the role of claudin isoforms in genitourinary cancer as a prognostic marker. The review provides two tables that list the changes observed in the abundances of various claudin isoforms and their prognostic significance. Claudins are the largest family of integral cell junction proteins, consisting of 27 known isoforms (Liu et al., 2016), which differ in their functions and tissue specificities. It is not surprising, therefore, that the prognostic picture emerging from the tables is complex and sometimes contradictory.
